# Photochromic Polyurethanes Showing a Strong Change of Transparency and Refractive Index

**DOI:** 10.3390/polym9090462

**Published:** 2017-09-20

**Authors:** Luca Oggioni, Chiara Toccafondi, Giorgio Pariani, Letizia Colella, Maurizio Canepa, Chiara Bertarelli, Andrea Bianco

**Affiliations:** 1 INAF—Osservatorio Astronomico di Brera, via Bianchi 46, 23807 Merate, Italy; luca.oggioni@brera.inaf.it (L.O.); giorgio.pariani@brera.inaf.it (G.P.); letizia.colella@brera.inaf.it (L.C.); 2 Dipartimento di Chimica, Materiali ed Ingegneria Chimica, Politecnico di Milano, p.zza L. Da Vinci 32, 20133 Milano, Italy; chiara.bertarelli@polimi.it; 3 OPTMATLAB, Dipartimento di Fisica, Università degli Studi di Genova, Via Dodecaneso 33, 16146 Genova, Italy; chiara.toccafondi5@gmail.com (C.T.); canepa@fisica.unige.it (M.C.); 4 Center for Nano Science and Technology, @PoliMi, Istituto Italiano di Tecnologia, Via Pascoli 70/3, 20133 Milano, Italy

**Keywords:** photochromism, diarylethenes, polyurethanes, refractive index, holography

## Abstract

Photochromic polymers have been studied as rewritable systems for optical elements with tunable transparency in the visible and refractive index in the NIR. Six diarylethene monomers have been synthesized to give thin films of photochromic polyurethanes. The absorption properties of the monomers in solution and of the corresponding polymeric films have been evaluated showing that a transparency contrast in the visible spectrum of the order of $10^{3}$ can be obtained by a suitable choice of the chemical structure and illumination wavelength. The change in the refractive index in the NIR have been determined by ellipsometry showing changes larger than $10^{-2}$. A trend of this variation with the absorption properties has been also highlighted. Fresnel lenses working on the basis of both a change of the transparency and the refractive index (amplitude and phase) have been demonstrated.

## 1. Introduction

Molecular species that change their properties as a consequence of an external well-defined stimulus are interesting in many fields. Indeed, smart materials can impart a new functionality to already-developed systems, or they can be applied to make simpler systems characterized by large degree of complexity. Photochromic materials belong to this class as they reversibly change color upon a light stimulus. For a long time, photochromic fulgides, spiropyrans, and spiroxazines have been used in transition lenses, which modify their transparency depending on the intensity of the sunlight [1]. Among photochromic materials, diarylethenes have a special role since they combine high photochromic performances with the thermal stability of the colored state [2]. The variation of both their extinction coefficient (in the UV–Vis spectral region) and their refractive index (in the NIR spectral region) has been applied to develop rewritable optical elements for optics [3,4] including amplitude elements such as computer generated holograms [5,6], transparency masks for lithography [7,8,9], and astronomical multiobject spectroscopy [10].

In order to be applied in optics, the photochromic substrate is required to: (i) achieve a large modulation of the target property; (ii) show a good photochromic response; and (iii) show good overall optical and mechanical properties. To achieve such goals, we proposed backbone photochromic polymers, where the photochromic units are part of the polymer main chain [11,12,13]. In particular, it has been shown that polyurethanes are good candidates, showing good resistance to solvents and allowing for versatile polymerization and processing. Moreover, polyurethanes (PUs), allowed for tunable properties, and high contrast of the processed films [14].

In this paper, we report on the synthesis and characterization of new photochromic polyurethanes based on hydroxyl-functionalized dithienyl- and ditiazolyl-ethenes. The performances in terms of transparency contrast in the visible and refractive index modulation are reported and discussed as a function of the chemical structure. Contrasts larger than 10^3^ can be easily reached, making possible the realization of efficient amplitude holograms for the visible spectral range. As for the refractive index, changes larger than 10^−2^ are obtained far from the resonance in the NIR.

## 2. Materials and Methods

**Figure 1:**
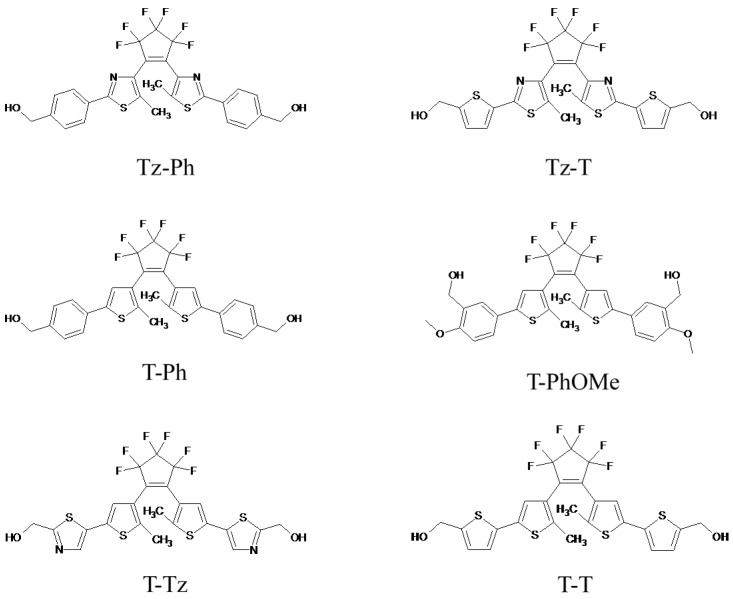
Chemical structure of the photochromic monomers.

Six photochromic monomers, which differ in the nature of the aromatic rings linked to the central perfluorocyclopentenes, have been synthesized to investigate their effect on the photochromic features of the resulting polymers (Figure 1). All the monomers are capped with hydroxymethylene to allow for the polymerization with the 4,4′-diisocyanate dicyclohexylmethane (H12MDI).

In the case of the monomer phenyl-terminated (**T-Ph**), the hydroxymethylene was introduced in two different positions and a methoxy group was added in the para-position (**T-PhOMe**) to study the influence of a donor group.

### 2.1. Synthesis of the Monomers

All the reagents have been purchased from Sigma-Aldrich Co. (St. Louis, MO, USA) and they have been used as received unless otherwise stated.

**Scheme 1:**
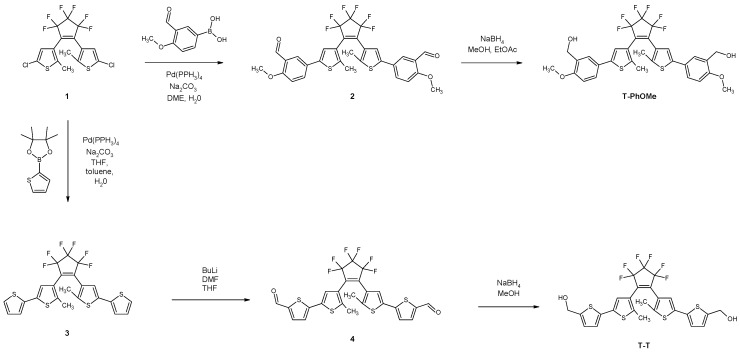
Synthesis of the monomers **T-PhOMe** and **T-T**.

1,2-Dithienylethenes were synthesized by Suzuki coupling between 1,2-bis-(5-chloro-2-methyl-3-thienyl)hexafluorocyclopentene (**1**) and a specific boronic acid or ester [15], followed by the formylation (if required) and the reduction of the aldehyde to the corresponding hydroxy-functionalized monomer (Scheme 1). Details of the synthesis of **T-Ph** are reported in reference 15.

#### 1,2-Bis-[5-*p*-methoxy-*m*-formylphenyl-3-thienyl]hexafluorocyclopentene (**2**)

**1** (0.90 g, 2.06 mmol,), 3-formyl-4-methoxyphenylboronic acid (0.93 g, 5.15 mmol), Na_2_CO_3_·10H_2_O (3.50 g, 12.35 mmol), Pd(PPh_3_)_4_ (0.23 g, 0.20 mmol), Dimethoxyethane (DME, 48 mL, degassed) and water (12 mL, degassed) were subsequently added to a reaction flask and stirred under inert atmosphere. The solution was refluxed under argon. After 24 h, 3-formyl-4-methoxyphenylboronic acid (0.18 g) and Pd(PPh_3_)_4_ (60 mg) were added and heating was continued for further 24 h. The organic phase was then extracted with Dichloromethane (DCM). The combined organic phase was dried (Na_2_SO_4_), filtered, and the solvent was evaporated under reduced pressure. Before purification, the solution was exposed to visible light to convert the material to the transparent form. Crystals of the desired compound **2** (1.02 g, 80% yield) were obtained after purification by column chromatography on silica gel (petroleum ether:ethyl acetate, 2:1 to 1:1 *v*/*v*).

^1^H NMR (400 MHz, CDCl_3_): δ 10.486 (s, 2H; –CHO), 7.98 (d, 2H, 4J(H,H) = 2.34 Hz; –H phenyl), 7.71 (dd, 2H, 3J(H,H) = 8.59 Hz, ^4^J(H,H) = 2.34 Hz; –H phenyl), 7.21 (s, 2H; –H thioph), 7.03 (d, 2H, ^3^J(H,H) = 8.586 Hz; –H phenyl), 3.97 (s, 6H; –OCH3), 1.98 (s, 6H; –CH3) ppm. HRMS (ESI): *m*/*z* calcd for C_31_H_22_F_6_O_4_S_2_ –H^+^: 635, [M − H^+^]; found 634.8, (M–).

#### 1,2-Bis-[5-*p*-methoxy-m-hydroxymethylphenyl-3-thienyl]hexafluorocyclopentene (**T-PhOMe**)

According to [16], NaBH_4_ (95 mg, 2.50 mmol) was slowly portionwise added to an ice-cooled solution of 2 (0.53 g, 0.83 mmol) in 100 mL methanol/ethyl acetate (1:1 *v*/*v*). The mixture was stirred for 2 h, and iced water was then added to quench the reaction. Solvent was removed under reduced pressure and the product was then extracted with ethyl acetate/water and the organic layer dried over Na_2_SO_4_. The crude was purified by flash chromatography with 1:1 petroleum ether/ethyl acetate as eluent to afford 0.30 g of the desired product as a white powder in 75% yield.

^1^H NMR (400 MHz, CDCl_3_): δ 7.46 (d, 2H, ^4^J(H,H) = 2.34 Hz; –H phenyl), 7.43 (dd, 2H, ^3^J(H,H) = 8.39 Hz, ^4^J(H,H) = 2.34 Hz; –H phenyl), 7.14 (s, 2H; –H thioph), 6.88 (d, 2H, ^3^J(H,H) = 8.58 Hz; –H phenyl), 4.70 (s, 4H; –CH_2_OH) 3.89 (s, 6H; –OCH_3_), 1.97 (s, 6H; –CH_3_) ppm. HRMS (ESI): *m*/*z* calcd for C_31_H_26_F_6_O_4_S_2_ –H^+^: 639, [M − H^+^]; found 638.9, (M–).

#### 1,2-Bis-[2-methyl-5-(2′-thienyl)-3-thienyl]hexafluorocyclopentene (**3**)

Thiophene 2-boronic acid pinacol ester (0.720 g, 3.43 mmol, Sigma-Aldrich Co., St. Louis, MO, USA), **1** (0.5 g, 1.14 mmol), Na_2_CO_3_·10H_2_O (1.43 g, 5.00 mmol) and Pd(PPh_3_)_4_ (0.300 g, 0.26 mmol) were dissolved in previously-degassed toluene (10 mL), tetrahydrofuran (THF, 10 mL), and water (5 mL). The reaction mixture was refluxed under argon for 24 h, then thiophene 2-boronic acid pinacol ester (0.270 g, 1.28 mmol) and Pd(PPh_3_)_4_ (0.200 g, 0.17 mmol) were added and the mixture was refluxed. The solution was extracted with diethyl ether and water, and the combined organic layers were dried over Na_2_SO_4_. Purification by flash chromatography on silica gel (petroleum ether:CH_2_Cl_2_ 9:1) afforded, after recrystallization from petroleum ether, compound **3** (425 mg, 69% yield).

^1^H NMR (400 MHz, CDCl_3_): d 7.23 (dd, 2H, ^3^J(H,H) = 5.086 Hz, ^4^J(H,H) = 0.803 Hz), 7.13 (dd, 2H, ^3′^J(H,H) = 3.75 Hz, ^4^J(H,H) = 0.803 Hz), 7.12 (s, 2H), 7.01 (dd, 2H, ^3^J(H,H) = 5.086 Hz, ^3′^J(H,H) = 3.748 Hz), 1.98 (s, 6H) ppm.

#### 1,2-Bis-[2-methyl-5-(5′-formyl-2′-thienyl)-3-thienyl]hexafluorocyclopentene (**4**)

To a stirred solution of **3** (0.320 g, 0.60 mmol) in anhydrous THF (15 mL), *n*-butyllithium (0.83 mL, 1.6 M in hexane, 1.32 mmol) was added dropwise at room temperature under an argon atmosphere. After 10 min, DMF was slowly added, and the reaction stirred for an additional 2 h. The solution was poured into H_2_O (5 mL)/HCl (5 mL) and extracted with diethyl ether. The organic layer was washed with H_2_O/NaHCO_3_ and dried over Na_2_SO_4_. Purification by flash chromatography on silica gel (petroleum ether:CH_2_Cl_2_ 1:2) afforded compound **4** as a blue–green powder (206 mg, 58% yield). ^1^H NMR (400 MHz, CDCl_3_): d 9.87 (s, 2H), 7.67 (d, 2H, ^3^J(H,H) = 4.015 Hz), 7.28 (s, 2H), 7.21 (d, 2H, ^3^J(H,H) = 4.015 Hz), 2.03 (s, 6H) ppm. 

#### 1,2-Bis-[2-methyl-5-(5′-hydroxylmethyl-2′-thienyl)-3-thienyl]hexafluorocyclopentene (**T-T**)

Compound **T-T** was synthesized with the same procedure described for compound **T-PhOMe**, starting from **4**. After purification by flash chromatography (ETP:EtOAc 1:2), 102 mg of a blue powder were obtained, in 67% yield.

^1^H NMR (400 MHz, CDCl_3_): d 7.08 (s, 2H), 6.99 (d, 2H, ^3^J(H,H) = 3.508 Hz), 6.91 (d, 2H, ^3^J(H,H) = 3.508 Hz), 4.80 (s, 4H), 1.97 (s, 6H) ppm.

**Scheme 2:**
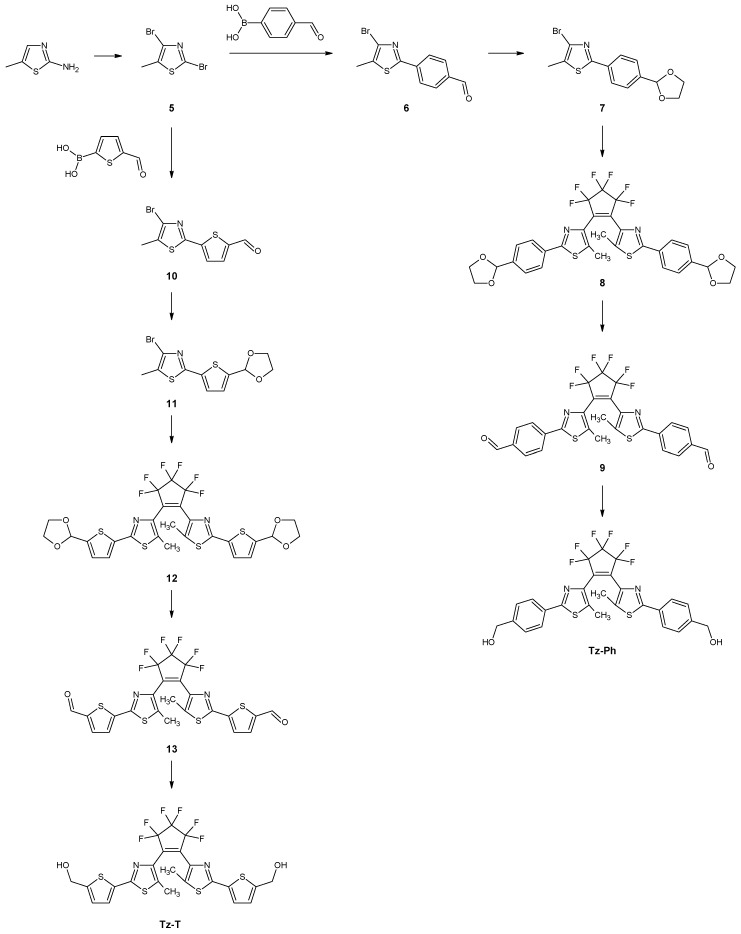
Synthesis of the monomers **Tz-T** and **Tz-Ph**.

**Tz-Ph** and **Tz-T** were synthesized starting from the Suzuki coupling of the di-bromothiazole (**5**) with a formyl benzene boronic acid and formyl thiophen boronic acid, respectively. The formyl group of the resulting products (i.e., 6 and 10) was first protected to form the 1,3-dioxalane, then a Dixon reaction with perfluorocyclopentene was carried out to afford the diarylethenes (**8**) and (**12**), which were deprotected and finally reduced with NaBH_4_ (see Scheme 2).

#### 2,4-Dibromo-5-methylthiazole (**5**)

A solution of 3.8 g of sodium nitrite in 8 mL of water was slowly added to a stirred solution of 2-amino-5-methylthiazole (2 g, 17.5 mmol) in 20 mL of H_3_PO_4_ and 10 mL of nitric acid at 0 °C. After 20 min the reaction was dropwise added to a solution of 2.6 g of copper(I) bromide in 19 mL of HBr. After four hours the reaction mixture was poured into aq NaOH, followed by the addition of NaHCO_3_ until reaching neutrality. The mixture was then extracted with ethyl acetate, and washed with sodium thiosulfate and water. The organic layer was dried over Na_2_SO_4_ and the solvent removed under reduced pressure to give 3.3 g of the desired product (**5**) in 73% yield.

^1^H NMR δ (400 MHz, ppm in CDCl_3_): 2.35 (s, 3H, –CH_3_).

#### 4-Bromo-5-methyl-2-*p*-formylphenylthiazole (**6**)

2,4-Dibromo-5-methylthiazole **5** (2.0 g, 7.78 mmol) and Pd(PPh_3_)_4_ (909 mg, 0.788 mmol) were dissolved in 128 mL of degassed DME. 4-Formyl-benzen boronic acid (1.06 g, 7.09 mmol) and 64 mL of 1 M aqueous solution of Na_2_CO_3_ were subsequently added to the reaction mixture under stirring. After 20 h under reflux, the reaction mixture was extracted with DCM/water and the organic layer is dried over Na_2_SO_4_. After solvent removal, flash chromatography using hexane:ethyl acetate 9:1 as eluent afforded 1.63 g of the desired product in 73% yield.

^1^H NMR δ (400 MHz, ppm in CDCl_3_): 10.05 (s, 1H, –CHO), 8.04 (d, 2H, ^3^J(H,H) = 8.4 Hz, Ph–H), 7.93 (d, 2H, ^3^J(H,H) = 8.4 Hz, Ph–H), 2.48 (s, 3H, –CH_3_).

#### 4-Bromo-5-methyl-2-[*p*-(1,3-dioxolane)phenyl]thiazole (**7**)

**6** (680 mg, 2.4 mmol), ethylene glycol (1.66 mL, 29.7 mmol), and *p*-toluene sulfonic acid monohydrate (15.6 mg, 0.24 mmol) were dissolved in 26 mL of toluene and refluxed overnight. The reaction mixture was cooled down to room temperature, poured into 10% aq. NaOH, extracted with DCM/water and dried over Na_2_SO_4_. After solvent removal, flash chromatography using hexane:ethyl acetate (ratio from 9:1 to 7:3) afforded 713 mg of **7** as a white powder in 91% yield. ^1^H NMR δ (400 MHz, ppm in CDCl_3_): 7.89 (d, 2H, ^3^J(H,H) = 8.2 Hz, Ph–H), 7.53 (d, 2H, ^3^J(H,H) = 8.2 Hz, Ph–H), 5.85 (s, 1H, –CH), 4.09 (m, 4H, O–CH_2_), 2.45 (s, 3H, –CH_3_).

#### 1,2-Bis-[2-methyl-5-(*p*-(1,3-dioxolane)phenyl)-thiazol-3-yl]perfluorocyclopentene (**8**)

*n*-BuLi (1.4 mL, 2.5 M in *n*-hexane) was added dropwise to a stirred solution of **7** (1.1 g, 3.37 mmol) in 40 mL anhydrous THF at −78 °C. After 15 min, 230 μL of perfluorocyclopentene were added. The reaction mixture was allowed to warm to room temperature, poured into water, extracted with DCM, and dried over Na_2_SO_4_. After solvent removal, the raw product was purified by flash chromatography with hexane:ethyl acetate (6:4) as eluent to afford 400 mg of the desired product **8** in 36% yield. The reaction provided also 700 mg of the monosubstituted product. ^1^H NMR δ (400 MHz, ppm in CDCl_3_): 7.86 (d, 4H, ^3^J(H,H) = 8.3 Hz, Ph–H), 7.52 (d, 4H, ^3^J(H,H) = 8.3 Hz, Ph–H), 5.85 (s, 2H, –CH), 4.08 (m, 8H, O–CH_2_), 2.13 (s, 6H, –CH_3_). Monosubstiuted product. ^1^H NMR δ (400 MHz, ppm in CDCl_3_): 7.90 (d, 2H, ^3^J(H,H) = 8.3 Hz, Ph–H), 7.53(d, 2H, ^3^J(H,H) = 8.3 Hz, Ph–H), 5.85 (s, 1H, –CH), 4.08 (m, 4H, O–CH_2_), 2.5 (s, 3H, –CH_3_).

#### 1,2-Bis-[2-methyl-5-(*p*-formyl)-phenyl-thiazol-3-yl]perfluorocyclopentene (**9**)

**8** (400 mg, 0.6 mmol) was dissolved in 30 mL of hot acetone, then *p*-toluenesulfonic acid pyridine salt (603 mg, 2.4 mmol) was added. The reaction mixture was refluxed for 8 h, poured into water, and extracted with diethyl ether, and the organic layer dried over Na_2_SO_4_. After solvent removal, 347 mg of the desired product were obtained as white/yellow powder in quantitative yield. ^1^H NMR δ (ppm in CDCl_3_): 10.05 (s, 2H, –CHO), 8.01 (d, 4H, ^3^J(H,H) = 8.3 Hz, Ph–H), 7.92 (d, 4H, ^3^J(H,H) = 8.3 Hz, Ph–H), 2.21 (s, 6H, –CH_3_).

#### 1,2-Bis-[2-methyl-5-(*p*-hydroxymethyl)-phenyl-3-thiazolyl]perfluorocyclopentene (**Tz-Ph**)

NaBH_4_ (57 mg, 1.51 mmol) was added to a solution of **9** (345 mg, 0.6 mmol) in 70 mL of ethyl acetate and 35 mL of methanol, and stirred at 0 °C. After two hours the solution was concentrated, the residue was washed with ethyl acetate and filtered. After solvent removal, the filtrate gave a dark yellow crystalline powder in quantitative yield. ^1^H NMR δ (400 MHz, ppm in CDCl_3_): 7.84 (d, 4H, ^3^J(H,H) = 8.0 Hz, Ph–H), 7.40 (d, 4H, ^3^J(H,H) = 8.2 Hz, Ph–H), 4.73 (s, 4H, –CH_2_), 2.15 (s, 6H, –CH_3_).

#### 4-Bromo-5-methyl-2-*p*-formylthienylthiazole (**10**)

2,4-Dibromo-5-methylthiazole 5 (3.23 g, 12.57 mmol) and Pd(PPh_3_)_4_ (1.76 g, 11.32 mmol) were dissolved in 230 mL of degassed DME. 5-Formyl-thiophene boronic acid (1.75 g, 11.32 mmol) and 115 mL of 1 M aqueous solution of Na_2_CO_3_ were subsequently added to the reaction mixture under stirring. After overnight reflux, the reaction mixture was extracted with DCM/water and the organic layer is dried over Na_2_SO_4_. After solvent removal, flash chromatography using hexane:ethyl acetate 9:1 as an eluent afforded 1.30 g of the desired product **10** as a pale yellow solid in 36% yield. ^1^H NMR δ (400 MHz, ppm in CDCl_3_): 9.92 (s, 1H, –CHO), 7.69 (d, 1H, ^3^J(H,H) = 4.09 Hz, Th–H), 7.52 (d, 1H, ^3^J(H,H) = 3.90 Hz, Th–H), 2.46 (s, 3H, –CH_3_).

#### 4-Bromo-5-methyl-[5-(1,3-dioxolane)thien-2-yl]thiazole (**11**)

**10** (1.29 g, 4.48 mmol), ethylene glycol (3.1 mL, 55.5 mmol) and *p*-toluene sulfonic acid monohydrate (85.6 mg, 0.45 mmol) were dissolved in 50 mL of toluene and refluxed overnight. The reaction mixture was cooled to room temperature, poured into water, extracted with DCM/water and dried over Na_2_SO_4_. After solvent removal, flash chromatography using hexane:ethyl acetate (ratio from 8.5:1.5) afforded 1.13 g of **11** as a white powder in 76% yield.

^1^H NMR δ (400 MHz, ppm in CDCl_3_): 7.33 (d, 1H, ^3^J(H,H) = 3.7 Hz, Th–H), 7.08 (d, 1H, ^3^J(H,H) = 3.7 Hz, Th–H), 6.11 (s, 1H, –CH), 4.02 (m, 4H, O–CH_2_), 2.40 (s, 3H, –CH_3_).

#### 1,2-Bis-[2-methyl-(5-(1,3-dioxolane)thien-2-yl)-3-thiazolyl]perfluorocyclopentene (**12**)

*n*-BuLi (1.48 mL, 2.5 M in *n*-hexane) was added dropwise to a stirred solution of **11** (1.20 g, 3.6 mmol) in 40 mL anhydrous THF at −78 °C. After 15 min, 240 μL of perfluorocyclopentene were added. The reaction mixture was allowed to warm to room temperature, poured into water, extracted with DCM, and dried over Na_2_SO_4_. After solvent removal, the raw product was purified by flash chromatography with hexane:ethyl acetate (ratio from 8:2 to 6:4) as an eluent to afford 70 mg of the desired product 12 in 5% yield. The reaction provided also 758 mg of the monosubstituted product. ^1^H NMR δ (400 MHz, ppm in CDCl_3_): 7.31 (d, 2H, ^3^J(H,H)J = 3.90 Hz, Th–H), 7.07 (d, 2H, ^3^J(H,H) = 3.90 Hz, Th–H), 6.10 (s, 2H, –CH), 4.05 (m, 8H, O–CH_2_), 2.14 (s, 6H, –CH_3_). Monosubstituted product: ^1^H NMR δ (400 MHz, ppm in CDCl_3_): 7.36 (d, 1H, ^3^J(H,H)J = 3.70 Hz, Th–H), 7.10 (d, 1H, ^3^J(H,H) = 3.70 Hz, Th–H), 6.11 (s, 1H, –CH), 4.08 (m, 4H, O–CH_2_), 2.5 (s, 3H, –CH_3_).

#### 1,2-Bis-[2-methyl-(5-formylthien-2-yl)-3-thiazolyl]perfluorocyclopentene (**13**)

**12** (350 mg, 0.54 mmol) was dissolved in 30 mL of acetone, then *p*-toluenesulfonic acid pyridine salt (677 mg, 3.9 mmol) was added. The reaction mixture was refluxed for 8 h, poured into water, and extracted with DCM, and the organic layer dried over Na_2_SO_4_. After solvent removal, 315 mg of the desired product **13** were obtained as yellowish powder in quantitative yield. ^1^H NMR δ (400 MHz, ppm in CDCl_3_): 9.90 (s, 2H, CHO), 7.68 (d, 2H, ^3^J(H,H) = 3.7 Hz, Th–H), 7.48 (d, 2H, ^3^J(H,H) = 3.7 Hz, Th–H), 2.31 (s, 6H, –CH_3_).

#### 1,2-Bis-[2-methyl-(5-hydroxymethyl-thien-2-yl)-3-thiazolyl]perfluorocyclopentene (**Tz-T**)

NaBH_4_ (56 mg, 1.48 mmol) was added to a solution of 13 (315 mg, 0.53 mmol) in 70 mL of ethyl acetate and 35 mL of methanol, stirred at 0 °C. After two hours the solution was concentrated, the residual was washed with ethyl acetate and filtered. After solvent removal, the filtrate gave a dark yellow crystalline powder in quantitative yield.

^1^H NMR δ (400 MHz, ppm in CDCl_3_): 7.30 (d, 2H, ^3^J(H,H) = 3.3 Hz, Th–H), 6.94 (d, 2H, ^3^J(H,H) = 3.3 Hz, Th–H), 4.80 (s, 4H, –CH_2_), 2.19 (s, 6H, –CH_3_).

**Scheme 3:**
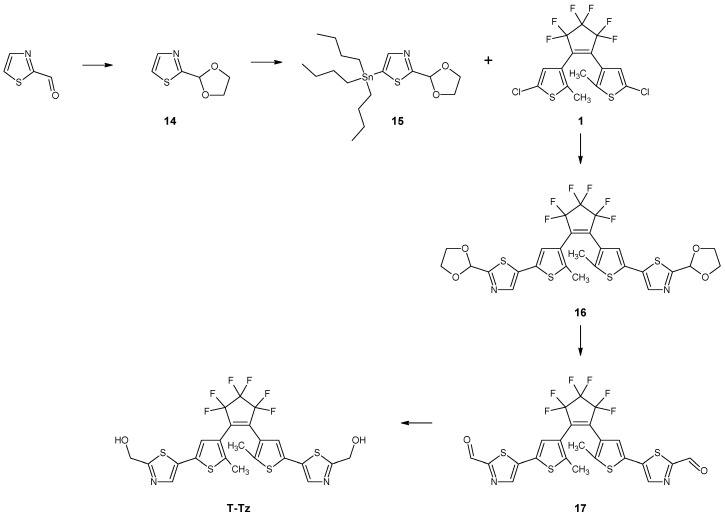
Synthesis of the monomer **T-Tz**.

**T-Tz** was obtained by Stille cross-coupling between the tributyl stannate (**15**) and the 1,2-bis-(5-chloro-2-methyl-3-thienyl)hexafluorocyclopentene (**1**). The 1,3-dioxane of the obtained diarylethene (**16**) was first deprotected and the resulting formyl group was reduced, as previously reported for the other species (see Scheme 3).

#### 2-Thiazolyl-1,3-dioxolane (**14**)

2-Formylthiazole (1.8 g, 15.9 mmol), 11.1 mL of ethylene glicole, and 302 mg of *p*-toluensulfonic acid monohydrate were dissolved in 60 mL of toluene and refluxed under stirring for two hours. The reaction mixture was extracted with ethyl acetate/water. After solvent removal, the raw product was purified by flash chromatography using petroleum benzene:ethyl acetate 8.5:1.5 to afford 1.96 g of the desired product **14** in 79% yield.

^1^H NMR δ (400 MHz, ppm in CDCl_3_): 7.81 (d, 1H, ^3^J(H,H) = 3.30 Hz, Tz–H), 7.36 (d, 1H, ^3^J(H,H) = 3.30 Hz, Tz–H), 6.16 (s, 1H, –CH), 4.12 (m, 4H, O–CH_2_).

#### Tributyl-5-(1,3-dioxolane)-thiazol-2-yl stannate (**15**)

5.4 mL of *n*-BuLi (2.5 M in *n*-hexane) was added dropwise to a stirred solution of 14 (1.78 g, 11.3 mmol) in 80 mL anhydrous THF at −78 °C. After 20 min, 3.7 mL of tributyl tin chloride were added. The reaction mixture was stirred at −78 °C for one hour, then it was allowed to warm to room temperature, quenched with water, extracted with ethyl acetate and dried over Na_2_SO_4_. After solvent removal, the raw product was purified by flash chromatography with petroleum benzine:ethyl acetate (9:1) as an eluent to afford 4.43 g of the desired product **15** in 88% yield.

^1^H NMR δ (400 MHz, ppm in CDCl_3_): 7.73 (s, 1H, Tz–H), 6.21 (s, 1H, –CH), 4.14 (m, 4H, O–CH_2_), 1.55 (m, 6H, –CH_2_–), 1.34 (m, 6H, –CH_2_–), 1.14 (t, 6H, ^3^J(H,H) = 8.07 Hz, Sn–CH_2_), 0.90 (t, 9H, ^3^J(H,H) = 7.33 Hz, –CH_3_).

#### 1,2-Bis-[2-methyl-5-((1,3-dioxolane)-thiazol-2-yl)-3-thienyl]perfluorocyclopentene (**16**)

1,2-Bis-(2-methyl-5-chloro-3-thienyl)perfluorocyclopentene (500 mg, 1.14 mmol), 15 (1.02 g, 2.29 mmol), Pd(PPh3)4 (264 mg, 0.228 mmol) were refluxed under argon for 24 h. The raw product was purified by flash chromatography using hexane:ethyl acetate (ratio from 7:3 to 6:4) to afford 490 mg of the desired product **16** in 63% yield.

^1^H NMR δ (400 MHz, ppm in CDCl3): 7.79 (s, 2H, Tz–H), 7.11 (s, 2H, Th–H), 6.10 (s, 2H, –CH), 4.13 (m, 8H, O–CH_2_), 2.00 (s, 6H, –CH_3_).

#### 1,2-Bis-[2-methyl-5-(4-formyl-thiazol-2-yl)-3-thienyl]perfluorocyclopentene (**17**)

**16** (460 mg, 0.71 mmol) was dissolved in 40 mL of acetone, then *p*-toluenesulfonic acid pyridine salt (1.29 g, 5.13 mmol) was added. The reaction mixture was refluxed for 24 h, poured into water and extracted with ethyl acetate, and the organic layer dried over Na_2_SO_4_. After solvent removal, flash chromatography using hexane:ethyl acetate 8:2 as an eluent afforded 403 mg of the desired product **17** were obtained as yellowish powder in 97% yield.

^1^H NMR δ (400 MHz, ppm in CDCl_3_): 9.94 (s, 2H, CHO), 8.09 (s, 2H, Tz–H), 7.29 (s, 2H, Th–H), 2.06 (s, 6H, –CH_3_).

#### 1,2-Bis-[2-methyl-5-(4-hydroxymethyl-thiazol-2-yl)-3-thienyl]perfluorocyclopentene (**T-Tz**)

NaBH_4_ (58 mg, 1.52 mmol) was added to a solution of **17** (360 mg, 0.61 mmol) in 70 mL of ethyl acetate and 35 mL of methanol, stirred at 0 °C. After two hours the solution was concentrated, the residual was washed with ethyl acetate and filtered. After solvent removal, the filtrate gave a dark yellow crystalline powder in quantitative yield.

^1^H NMR δ (400 MHz, ppm in CDCl_3_): 7.78 (s, 2H, Tz–H), 7.08 (s, 2H, Th–H), 5.01 (s, 4H, –CH_2_), 2,05 (s, 6H, –CH_3_).

### 2.2. Polyurethane Production

The general procedure to synthesize the diarylethene-based polyurethanes consists of the reaction between the specific photochromic monomer (in a 50% ratio on the total OH groups, which corresponds to about 40 wt % of all components), polyols, and 4,4′-diisocyanate dicyclohexylmethane (H12MDI), as described in the literature [14].

The films were obtained by spin coating on different substrates. Quartz substrates have been used for the UV–Vis characterization since quartz starts to absorb at wavelengths below 200 nm. Silicon substrates have been exploited for the ellipsometric measurements. The rotation speed of the spin-coater was varied between 750 to 4000 rpm to obtain films with thicknesses of 1000 nm and 300 nm, respectively. The starting solutions showed a concentration of 1 g of monomers (both dialcohol and diisocyanate) in 6 mL of solvent (butyl acetate for **T-Ph**, **Tz-Ph**, and **T-PhOMe**, and propylene glycol monomethyl ether acetate for **T-T**, **Tz-T**, and **T-Tz**).

### 2.3. UV–Vis Absorption Spectrsocopy

The UV–Vis spectra have been collected using a Jasco V570 spectrometer (JASCO International Co. Ltd., Tokyo, Japan) for both monomers in ethanol solution and thin films deposited on quartz substrates. For the solutions, knowing the concentration of the solution and applying the Lambert–Beer law, the spectra in terms of the molar extinction coefficient have been calculated. As for the films, the thickness has been evaluated and the molar extinction coefficient calculated knowing the relative concentration and setting a density of the polymer equal to 1.1 g/mL.

### 2.4. Spectroscopic Ellipsometry

Ellipsometry was employed to determine the film thickness and the refractive index modulation vs. photo-stimulation, similarly to a recent paper on a related system [17]. Measurements were performed in a darkened room at 60°, 65°, and 70° angles of incidence, using a rotating compensator instrument (M-2000, J.A. Woollam Co. Inc., Lincoln, NE, USA) equipped with a 75 W Xe lamp. The measurements covered the 245–1700 nm spectral interval. The spot size on the sample was approximately a few mm^2^. Each sample was measured first in its open-ring form using a UV filter to prevent conversion during the procedure; the sample was then converted into closed-ring form with an UV LED at 325 nm (power density of $150\;\mu W/{\mathrm{cm}}^{2}\;$for 10–15 min) and measured again. Dynamic measurements were collected in real-time during the conversion from the uncolored to the colored form. The data analysis exploited the manufacturer software (VASE, Lincoln, NE, USA). In this paper Ψ and Δ data have been fitted in the 800 nm < λ < 1700 nm range where both colored and uncolored forms are transparent. Cauchy and Sellmeier models were used for the wavelength dependence of the refractive index. In this spectral region, both models provide the same results.

### 2.5. Microscopy

A phase contrast microscope (equipped with U-DICR, Olympus, Shinjuku, Tokyo, Japan) has been used to collect the images with a 20× objective. For the pure amplitude images, the phase shift element has been removed.

### 2.6. Optical Tests

The focusing tests have been performed using a Fresnel lens with a designed focal length of 20 mm at 633 nm. The laser beam at 650 nm (for the amplitude hologram reconstruction) and at 980 nm (for pure phase hologram reconstruction) has been fiber-coupled. At the fiber exit, the beam has been collimated and directed to the diffractive optical element. A CMOS camera (DCC1545M, Thorlabs, Newton, NJ, USA) has been placed at the focal position, and then the lens moved out of focus to record the different images.

## 3. Results

### 3.1. Absorption Properties in the UV–Vis Spectral Range

**Figure 2:**
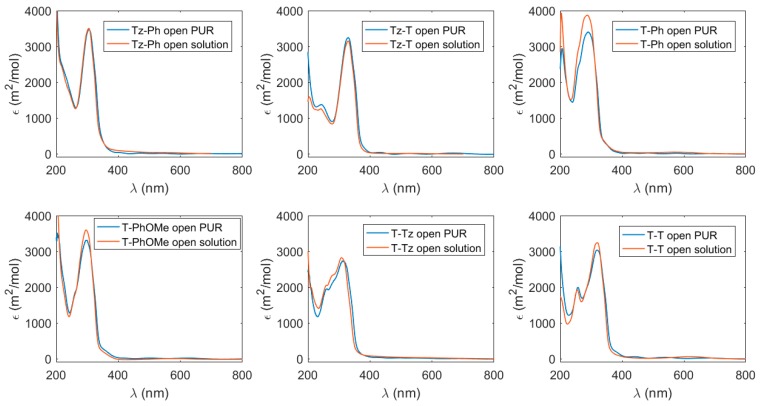
Molar extinction coefficients for the open-ring forms (uncolored) of the investigated monomers dissolved in ethanol (red line) and after polymerization (PU film, blue line).

**Figure 3:**
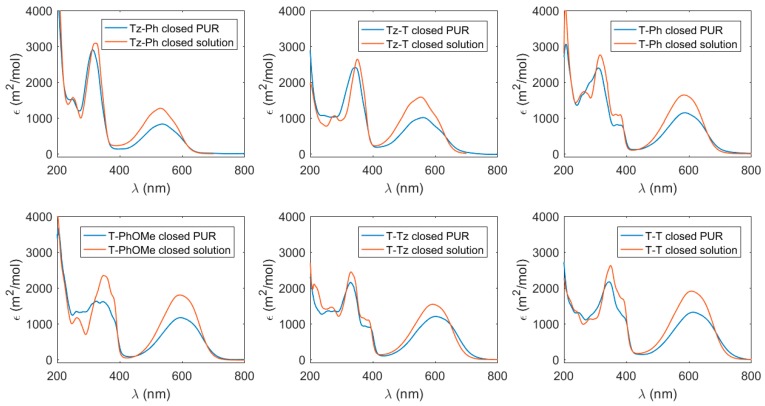
Molar extinction coefficients for the closed-ring forms (colored) of the investigated monomers dissolved in ethanol (red line) and after polymerization (PU film, blue line).

The absorption properties have been evaluated both for the dialcohol monomers in solution and for the corresponding PU thin films. The UV–Vis spectra in terms of the molar extinction coefficient are reported in Figure 2 and Figure 3 for both the open- and closed-ring forms, respectively.

For all the photochromic systems, the absorption spectra of the open form are very similar in solution and in polyurethane films, whereas a more significant difference occurs for the closed forms. Specifically, the absorption coefficient of polyurethane films are about the 68% of the value in the ethanol solution in the visible region, which is ascribed to both the different environment and a lower conversion degree of the photo-induced ring-closure at the solid state.

**Figure 4:**
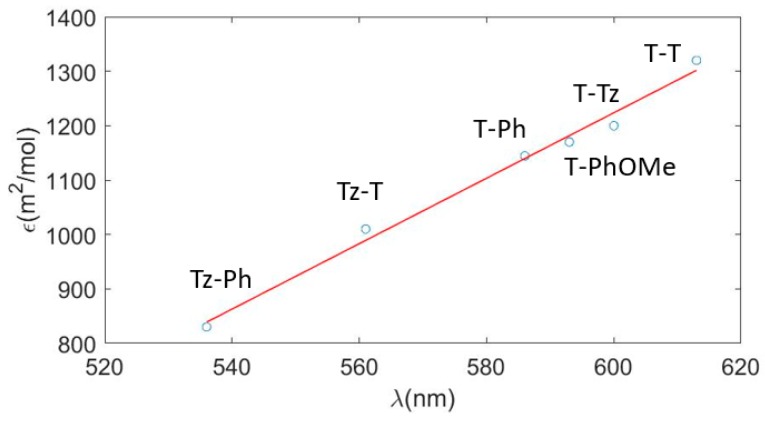
Plot of the maximum values of the molar extinction coefficient of the colored forms in the visible region vs. their spectral positions.

Interestingly, we noticed a linear dependence of the molar extinction coefficient and the peak wavelength in the visible for the colored form (Figure 4).

The position of absorption bands of diarylethene molecules in the closed ring form is related to their structure: molecules with higher degree of π conjugation show a redshift of their absorption spectrum and a rise of the corresponding intensity. It is known that thienyl leads to a more effective conjugation than the phenyl, due to both lower inter-ring torsional angles and less aromaticity. On the other hand, thiazole is blue-shifted with respect to thiophene because of a less effective conjugation length.

In order to predict the performance of PU films in terms of transparency change as a function of the film thickness we carried out simulations based on a kinetic model we recently developed [18]. This model allows for calculating the absorbance properties of a photochromic layer, simulating the degree of conversion of the photochromic unit upon illumination at a specific wavelength. For any specimen, we calculated the contrast parameter at the absorbance peak in the visible region, defined as follows [4]:

(1) $$C\;=\;\frac{T_{o}\left(\lambda \right)}{T_{c}\left(\lambda \right)}\;=\;\frac{1}{10^{-A\left(\lambda \right)}}$$

where *T_o_* and *T_c_* are the transmittance of the film in the uncolored (open-ring) and colored (closed-ring) forms at the specific wavelength. If we consider that the open form does not show any absorption in the visible, the contrast depends only on the transmittance (absorbance) of the colored form.

**Figure 5:**
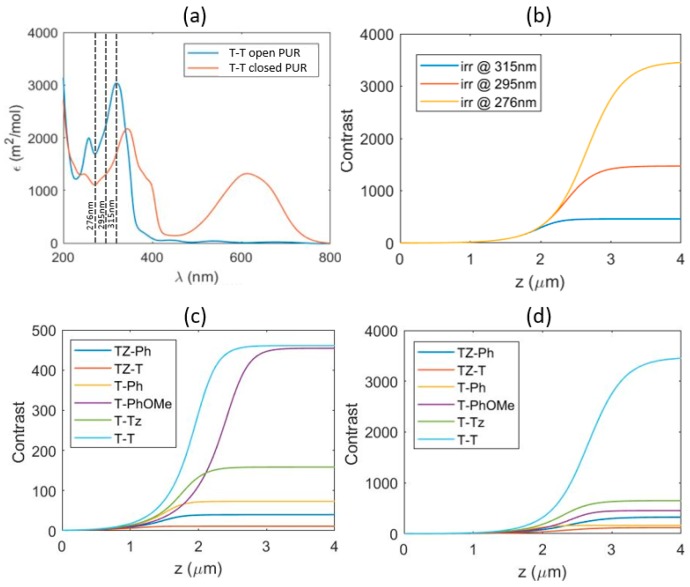
(**a**) Molar extinction coefficients for the open- and closed-ring forms of **T-T**-based PU. The dashed lines indicate the illumination wavelength considered, including the best (276 nm) and the worst (315 nm) conversion conditions. (**b**) Simulated contrast vs. the thickness of a polyurethane film for **T-T**-based PU. Simulated contrast vs. the thickness of the different photochromic polyurethanes (**c**), considering the wavelength of irradiation at the UV peaks of the open-ring form and (**d**) considering the best irradiation wavelength.

According to the Lambert–Beer law, the absorbance is a function of the concentration of the photochromic units and the film thickness. Moreover, the final absorbance of the film also depends on the degree of photochromic conversion actually achieved through the film thickness. As reported in Figure 2 and Figure 3, the absorption spectra of the photochromic PUs in the UV region are quite complex for both photochromic forms and the ratio of their extinction coefficients differs according to the wavelength we consider. To explain this issue, we report in Figure 5a, the absorption spectra of **T-T**-based PU films that were converted with three different wavelengths in the UV region. A larger absorption of the open form means a faster conversion rate; on the other hand, during the conversion, the closed form tends to absorb the incoming UV light according to the specific absorption at the illuminating wavelength. Therefore, a larger absorption of the closed form means a more difficult conversion of the material through the film thickness and eventually a lower degree of conversion.

The calculated contrast as a function of the film thickness is reported in Figure 5b for the **T-T**-based films. Below a threshold thickness of about 2 μm, no dependence on the illumination wavelength occurs. Indeed, the total absorbance of the film in the UV region is small and UV light is not strongly attenuated through the film, allowing for the total conversion. For thicker films, UV light is progressively absorbed inside the volume during the conversion and only a limited thickness of the photochromic film is converted. This penetration limit depends on the absorbance of the closed-ring form of the photochromic unit at the irradiation wavelength. Therefore, considering the **T-T**-based PU, the largest penetration depth and, hence, the maximum contrast, is achieved at a wavelength of 276 nm, where the residual absorbance of the closed form is the smallest. 

To highlight the effect of the selection of the irradiation line to perform the conversion to the colored form, in Figure 5c,d we report the calculated contrast of all the PU films herein considered when exposed at the wavelength corresponding to the absorption maximum of the open-ring form (c) and at the best condition for UV-penetration (d). It is worth noting that a larger contrast in the visible region is obtained when the absorption coefficient of the closed form in the visible is larger than the absorption coefficient of the closed form in the UV. This is the case of the **T-T**-based PU, which shows the highest ratio of ξ_c–vis_/ξ_c–UV_ and provides the best values of contrast among all PUs, as reported in Figure 5d.

### 3.2. Refractive Index Change in the NIR Spectral Range

**Figure 6:**
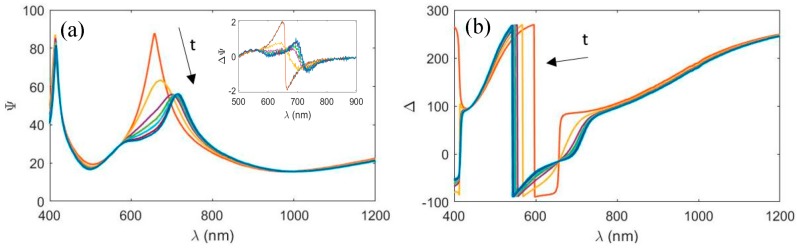
Plots of Ψ and Δ ((**a**,**b**), respectively) measured at different UV exposure times for a **T-T** polyurethane sample. The inset in (**a**) reports the difference spectra δΨ = Ψ(t) − Ψ(0) in the spectral region to emphasize the polymer color change.

The refractive index variation induced by the photochromic isomerization was determined by ellipsometry, which allows for a good accuracy of the measurement, since Ψ and Δ strongly vary during the conversion upon UV exposure from the uncolored to the colored forms (Figure 6).

We notice large Ψ and Δ changes during the illumination with UV light that converts the film from the uncolored to the colored forms. In particular, the most evident changes are located in the 600–800 nm spectral region, reflecting that the strong extinction change occurs when going from the uncolored to the colored form. This change is even clearer when looking at the inset plot of Figure 6a where the difference spectra δΨ = Ψ(t) − Ψ(0) are reported as a function of the wavelength.

In Table 1, we report the obtained values of the refractive index variation (Δ*n*) between the colored (closed) and the transparent (open) forms of the films at two different wavelengths: near the absorption band of the colored form in the visible (λ = 800 nm) and in the near infrared region (λ = 1700 nm). The values of Δ*n* are the average of the ellipsometric measurements performed on samples with different thicknesses. It is worth noting that such thicknesses (reported in the first column of Table 1) are larger than the usual thicknesses in common ellipsometric measurements and much larger than the thicknesses of photochromic films previously studied with the same approach [17].

**Figure 7:**
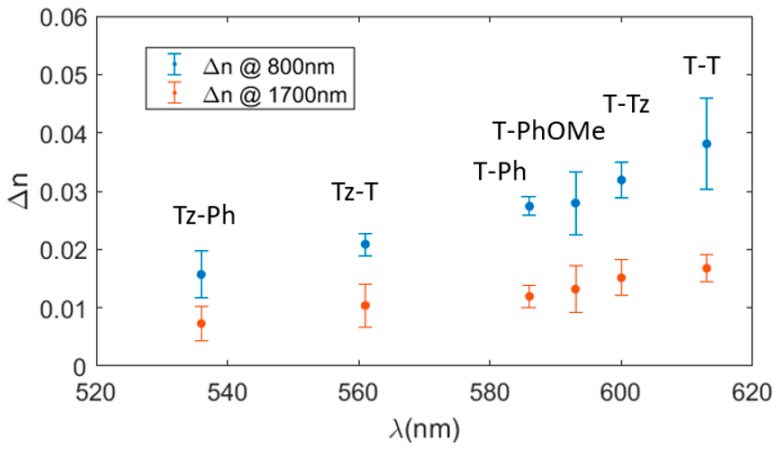
Δ*n* measured with ellipsometry at two different wavelengths for the polyurethanes under study. The results are plotted versus the peak position of the absorption band in the visible of the ring-closed form.

The change in the refractive index depends on the chemical structure of the photochromic moiety and it increases with the reduction in the wavelength due to the pre-resonance effect, as expected. Moreover, we notice that molecules with larger λ_peak_ in the visible and a larger extinction coefficient show a higher Δ*n* at any selected wavelength, even far from the resonance frequency (Figure 7).

To interpret these results, we consider the Sellmeier expression of the refractive index of dielectric materials as a function of the wavelength:

(2) $$n\left(\lambda \right)\;=\;\sqrt{1+\sum_{i}\frac{A_{i}\lambda^{2}}{\left(\lambda^{2}-\lambda_{i}^{2}\right)\;}}$$

The material is modelled as a collection of oscillators characterized by their strengths *A_i_* and their resonance wavelengths $\lambda_{i}$. Considering that the closed form of the photochromic PU shows an absorption in the visible spectrum, whereas the open form shows absorption only in the UV region, it is apparent that the Δ*n* increases at shorter wavelengths in the NIR region, since the refractive index of the closed form increases more steeply. This contribution is stronger for molecules showing absorption peaks at longer wavelengths in the visible. Another effect is due to the oscillator strength of the numerator, which is directly related to the intensity of the absorbance band. The larger the intensity, the larger the Δ*n*. Thanks to the correlation highlighted in Figure 4, this contribution adds to that of the position of the absorption band. Thus, we can conclude that photochromic molecules with a higher degree of conjugation in the ring-closed form not only provides a larger change in the transparency but also a higher value of Δ*n*.

### 3.3. Phase and Amplitude Patterns on PU Films

The large modulation of the transparency in the visible and the refractive index in the NIR for these photochromic polyurethanes has been exploited to make Fresnel patterns that behave as spherical lenses with defined focal lengths.

**Figure 8:**
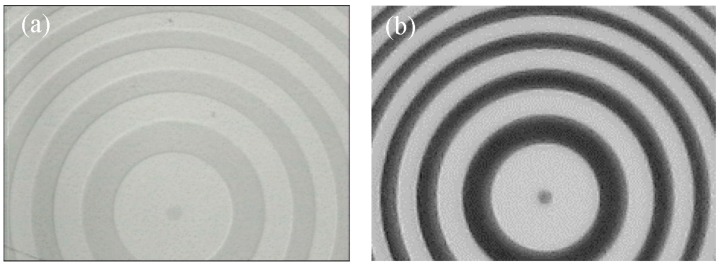
Images of a Fresnel lens written on a photochromic PU film (**T-Ph**). (**a**) An image collected with the phase-contrast microscope in order to highlight the phase modulation induced by the change of the refractive index; and (**b**) an image collected with a standard microscope where the variation of the transparency is enhanced.

In Figure 8, we show the microscope images of the pattern written by direct laser writing at 633 nm on a photochromic polyurethane film based on the molecule **T-Ph**. Figure 8a shows the image of the Fresnel pattern collected with a phase contrast microscope in order to highlight the phase change due to the difference in the refractive index. Such a phase change can also be a consequence of a variation in the local thickness due to the writing process; however, this effect has been avoided by optimizing the laser power, as previously reported [19]. Figure 8b shows the image collected with a standard microscope showing the pattern due to the different transparency of the colored and uncolored features. Clearly, the two patterns are exactly the same.

**Figure 9:**
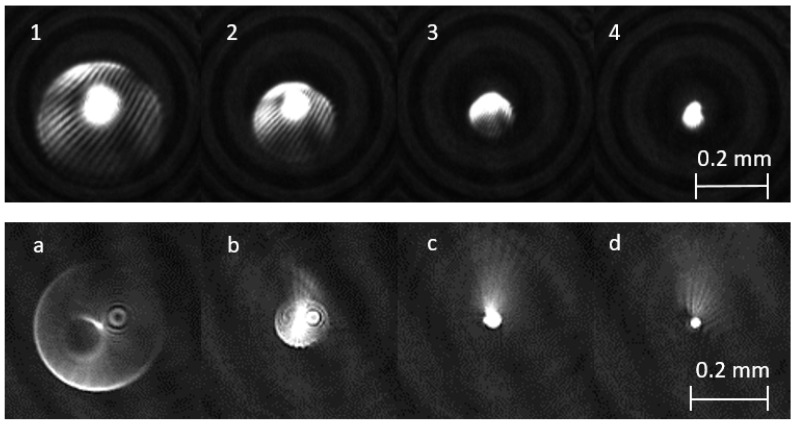
Images of the approaching steps to the focus point of a Fresnel lens written onto the photochromic film (**T-Ph**). The sequence from (**1**) to (**4**) refers to a laser in the visible region (λ = 650 nm), the sequence from (**a**) to (**d**) refers to a laser in the NIR (λ = 980 nm).

The Fresnel lens has been tested to demonstrate that it actually behaves as a spherical lens both in the visible region exploiting the contrast in absorption and in the NIR region exploiting the refractive index variation. In Figure 9, we report some steps of the focusing procedure of two lasers at 650 and 980 nm.

The focusing position is different between the two wavelengths and it is shorter (shorter focal length) at 980 nm in accordance to the diffraction grating equation. Indeed, fixing the spacing of the periodic structure, longer wavelengths are diffracted at larger angles, which becomes a shorter focal length in the case of a Fresnel spherical lens.

## 4. Conclusions

A set of photochromic polyurethanes based on diarylethene dialcohol monomers showing different chemical structures have been synthesized. Such films are extremely interesting as a rewritable and re-addressable platform for making amplitude and phase optical elements and holograms. Considering the change of the transparency in the visible spectrum, we showed how the contrast is strongly dependent not only on the chemical structure, but also on the UV wavelength used to convert the film to the colored form. Contrasts larger than 10^3^ are suitable for maximizing the efficiency of amplitude binary holograms, and can be easily achieved using the **T-T**-based PU films with a thickness on the order of 2.5 μm. Regarding the phase variation in the transparent region, namely λ > 800 nm, large changes of the refractive index have been determined by ellipsometry. We noticed that the Δ*n* increased approaching the visible spectral region because of a pre-resonance effect. We also highlighted a relationship between the Δ*n* and the position and intensity of the absorption band of the colored form in the visible. According to this relationship, we found that the **T-T**-based films showed the largest change with values approaching 0.04 at 800 nm. Such interesting and large refractive index modulation paves the way for a deeper analysis by spectral ellipsometry to obtain a detailed picture of the optical properties of the photochromic PU as a function of the electronic structure. The effectiveness of the amplitude and phase variation of the PU substrates has been demonstrated by realizing a Fresnel lens that focused the light both in the visible spectrum, based on the amplitude change, and in the NIR spectrum, based on the phase change.

## Figures and Tables

**Table 1:** Ellipsometric measurements of the film thickness and Δ*n* values at 800 and 1700 nm for films of photochromic polyurethanes.

PU	Thickness (nm)	Δ*n* at 800 nm × 10^−2^	Δ*n* at 1700 nm × 10^−2^
**Tz-Ph**	480	1.6	0.7
**Tz-T**	370,410,500	2.1	1.0
**T-Ph**	420,470,510	2.7	1.2
**T-PhOMe**	270,420	2.8	1.3
**T-Tz**	360,340	3.2	1.5
**T-T**	360,380,510	3.8	1.7

## References

[1] Crano J.C., Flood T., Knowles D., Kumar A., Van Gemert B. (1996). Photochromic compounds: Chemistry and application in ophthalmic lenses. Pure Appl. Chem..

[2] Irie M., Fukaminato T., Matsuda K., Kobatake S. (2014). Photochromism of diarylethene molecules and crystals: Memories, switches, and actuators. Chem. Rev..

[3] Spanò P., Zerbi F.M., Norrie C.J., Cunningham C.R., Strassmeier K.G., Bianco A., Blanche P.A., Bougoin M., Ghigo M., Hartmann P. (2006). Challenges in optics for Extremely Large Telescope instrumentation. Astron. Nachr..

[4] Bertarelli C., Bianco A., Castagna R., Pariani G. (2011). Photochromism into optics: Opportunities to develop light-triggered optical elements. J. Photochem. Photobiol. C.

[5] Alata R., Pariani G., Zamkotsian F., Lanzoni P., Bianco A., Bertarelli C. (2017). Programmable CGH on photochromic plates coded with DMD generated masks. Opt. Express.

[6] Pariani G., Bertarelli C., Dassa G., Bianco A., Zerbi G. (2011). Photochromic polyurethanes for rewritable CGHs in optical testing. Opt. Express.

[7] Andrew T.L., Tsai H.Y., Menon R. (2009). Confining Light to Deep Subwavelength Dimensions to Enable Optical Nanopatterning. Science.

[8] Menon R., Smith H.I. (2006). Absorbance-modulation optical lithography. J. Opt. Soc. Am. A.

[9] Pariani G., Castagna R., Menon R., Bertarelli C., Bianco A. (2013). Modeling absorbance-modulation optical lithography in photochromic films. Opt. Lett..

[10] Bianco A., Bertarelli C., Gallazzi M.C., Zerbi G., Giro E., Molinari E. (2005). Smart focal plane masks: Rewritable photochromic films for astronomical multi-object spectroscopy. Astron. Nachr..

[11] Stellacci F., Bertarelli C., Toscano F., Gallazzi M.C., Zotti G., Zerbi G. (1999). A high quantum yield diarylethene-backbone photochromic polymer. Adv. Mater..

[12] Bens A.T., Comanici R., Gabel B., Kryschi C., Martin H.D., Ritter H. (2003). Novel photosensitive polyurethanes based on photochromic dithienylethene monomers: Synthesis, characterization and photophysical properties of a new film-building material for photonic applications. e-Polymers.

[13] Wigglesworth T.J., Myles A.J., Branda N.R. (2005). High-content photochromic polymers based on dithienylethenes. Eur. J. Org. Chem..

[14] Pariani G., Castagna R., Dassa G., Hermes S., Vailati C., Bianco A., Bertarelli C. (2011). Diarylethene-based photochromic polyurethanes for multistate optical memories. J. Mater. Chem..

[15] Hermes S., Dassa G., Toso G., Bianco A., Bertarelli C., Zerbi G. (2009). New fast synthesis route for symmetric and asymmetric phenyl-substituted photochromic dithienylethenes bearing functional groups such as alcohols, carboxylic acids, or amines. Tetrahedron Lett..

[16] Sevez G., Gan J.A., Pan J.F., Sallenave X., Colin A., Saadoui H., Saleh A., Vogtle F., Pozzo J.L. (2007). Multi-addressable supramolecular gels based on linear amino acid and bisthienylcyclopentene. J. Phys. Org. Chem..

[17] Toccafondi C., Occhi L., Cavalleri O., Penco A., Castagna R., Bianco A., Bertarelli C., Comoretto D., Canepa M. (2014). Photochromic and photomechanical responses of an amorphous diarylethene-based polymer: A spectroscopic ellipsometry investigation of ultrathin films. J. Mater. Chem. C.

[18] Pariani G., Bianco A., Castagna R., Bertarelli C. (2011). Kinetics of Photochromic Conversion at the Solid State: Quantum Yield of Dithienylethene-Based Films. J. Phys. Chem. A.

[19] Pariani G., Bertarelli C., Bianco A., Schaal F., Pruss C. (2012). Characterization of photochromic computer-generated holograms for optical testing. Proc. SPIE.

